# Is Apicoectomy Ever Successful? If so, under What Conditions?

**Published:** 1922-07

**Authors:** Jos. D. Eby, Jos. H. Jaffer

**Affiliations:** New York City; New York City


### IS APICOECTOMY EVER SUCCESSFUL? IF SO, UNDER WHAT CONDITIONS?

A SYMPOSIUM. CONTRIBUTION FROM MAJ. JOS. D. EBY, D.D.S. AND CAPTAIN JOS. H. JAFFER, D.D.S., BOTH OF NEW YORK CITY, IN DENTAL ITEMS OF INTEREST, JUNE, 1922

It was our privilege to hear the papers read by Doctors Thomas P. Hinman and Harry B. Johnston before the First District Dental Society of New York in November, 1921, out of which this symposium has grown; also to have heard the discussions which followed.

There is nothing transpiring in any phase of dentistry which is of greater importance than this tremendous problem of analyzing and classifying the truths into a final solution of the effects and treatment of dento-alveolar disorders. This final solution will mark a new era of understanding by disclosing the facts so clearly as to undermine the wide range of variations in men's minds existing in a certain sense very deplorably today.

The extreme differences between that class who have become recognized by their radical extraction of all pulpless teeth, and the ultra-conservatives, must be admitted at the outset to be too great for either of these extremists to be right, and the truth, therefore, must exist somewhere between them.

After long years of contact and association with the brainiest of specialists in all branches of the healing art, we have come to the conclusion, inasmuch as it is a failing of the human mind to form beliefs best suited to individual liking, that the majority of conclusions reached and outspoken by those who are not qualified to speak, fall far short of the truth. In the particular subject in which we are now interested, this failing results from jumping at conclusions too quickly, or being led too easily by the influences of other minds, rather than from intensive study.

We would say, then, that ignorance in the question before us is the result of a lack of knowledge in such fundamental branches of science as biology, biochemistry, histology, physiology, pathology, bacteriology, with the association of internal medicine, therapeutics, surgery, etc., all of which must be intimately interwoven into a rational procedure sufficiently elastic to include within its scope the variations within each and every individual patient with whom we come in contact.

Since the time when the importance of the relation of foci of infection to general disease was established, shafts of knowledge budding from these various sources have now developed to where they have reached contact at a point which must normally be considered as the centroid of scientific facts, near the point where each radius has automatically converged from varying distances as governed by the relative importance of the various phases from whence they emanated.

If one or the other of these extremes, i. e., the radical or the ultraconservative, were situated in this center of scientific achievement then we would be confronted by the harassment of thousands of people by all of the complications and sequelæ attending the extraction of every pulpless tooth on the one hand as against the possibilities of being responsible for the undermining of health or possible death of many people from ultraconservative methods on the other hand. Long established facts, however, indicate that neither of these extreme methods of treatment is applicable in all cases but, instead, the treatment selected in each individual case should be determined by the condition of the tooth and the general physical condition of the patient.

### THE NOURISHMENT OF A TOOTH

It is an established fact that a tooth is developed and nourished through two important structures, i. e., the pulp and the peridental membrane, the pulp being responsible for the development of the enamel and dentin, the peridental membrane for the cementum and adjacent lamella of bone. Upon the completion of the development of the tooth the essential function of the pulp is to supply nourishment to the dentin and also sensation to the tooth.

The peridental membrane, however, continues to perform more important functions after the completion of development of the tooth in that it not only acts as a buffer between the tooth and surrounding bone and supplies the sense of touch to the tooth, but by virtue of the fact that it contains osteoblasts and osteoclasts on the outer surface with cementoblasts and cementoclasts on the inner surface, it is constantly causing changes in these structures to meet the demands of the varying local conditions.

When a tooth is deprived of its pulp it loses sensation and nourishment to the dentin, but the vitality of the cementum remains unchanged and the tooth retains its physiologic relationship with the adjoining tissues. Thus the fact is self-apparent that so long as the peri dental membrane remains intact, the tooth can not be considered to be a foreign body. When micro-organisms invade first the pulp canal, then the tissues remaining in the dentinal tubuli and finally penetrate beyond the tooth into the periapical tissues, the result is a conflict between the destruction of living tissues by the organisms as against the ingestion of organisms or their limitation to a certain area by the phagocytes.

The extent of this conflict is determined by the type and virulence of the organism on the one hand as against the great and varying forces of resistance of the individual on the other.

He who reckons without the keenest possible knowledge of variations of immunity is one who fails to summon his proper resources and it is in the finer interpretation of this point that the two extremists in this great subject must be called to answer why their empirical dogmas should be followed.

### THE FACTOR OF INDIVIDUAL IMMUNITY

The immunity of an individual must ultimately resolve itself down to the individual cell and in that form the question of both the quantitative and qualitative variations in biochemistry, not only in different individuals but within the same individual at different times must be taken as the basis of consideration. In the treatment of all diseases the aim should be to assist Nature to the extent required in returning the abnormal to normal, either therapeutically, surgically, or otherwise. The treatment of periapical disorders is no exception to this principle.

It therefore very naturally follows that if the resistance of an individual is subnormal, as manifested by constitutional disorders indicating that Nature's resources are too feeble to respond to any assistance, it is necessary to eliminate all infection as expeditiously as possible by tooth extraction, drainage, and curettement without any further consideration of more conservative treatment.

In cases of periapical infections where the resistance of the individual is normal, as manifested by the absence of all constitutional disorders, more conservative methods are to be considered. The principle factor governing the retention of teeth is the possibility of rendering the tooth and adjacent structures sterile, whether accomplished by direct medication, ionic medication, root resection, or any of their combinations.

After having seen the beautiful results obtained under the various methods of therapy and technic employed by such men as Doctors Buckley, Callahan, Hinman, Johnston, Grieves, Ottolengui, Rhein, Bannister and others, we believe that complete resolution and regeneration of bone is possible in rarefied areas which have resulted from infections around the apices of teeth. (Figures 1, 2 and 3.)

### ELECTROLYTIC MEDICATION

From recent conversations and careful examination of the works of Doctors Johnston, Hinman and Ottolengui, we are convinced that proper ionization with careful regard for the finest technic, is one of the most essential steps in the beginning of the treatment of pulpless teeth, regardless of what is to follow. It is a known fact that through electric ionization an escharotic as strong as 19 per cent. Churchill's iodin is broken up and transfused through an area so as to have a positive destructive effect on micro-organisms without serving as an irritant to the living cells.

Although it follows from the above that complete regeneration of bone is possible without further treatment than various medications and root canal fillings, surgical interference is advisable in certain selected cases.

Successful root resection is usually limited to single-rooted teeth, preferably the maxillary incisors, canines and less frequently the maxillary premolars and mandibular incisors. For reasons of inaccessibility, number of roots, etc., other teeth are out of consideration.

### CONDITIONS FAVORABLE TO APICOECTOMY

When there has been very little absorption of the alveolar border so that at least two-thirds of the peridental lamella is intact with vital peridental membrane attached, such teeth have been considered by rational minds to be good candidates for root resection.

Previous to entering into the surgical procedure of root resection, it is essential that proper root canal therapy and filling should be performed and preferably after thorough ionization. (Figures 4, 5 and 6.)

### BACTERIA IN THE TISSUES

There is very little evidence to support the sweeping statements of radical men who contend that, after re-entering the apparently healed and regenerated field in the periapical area, they have obtained positive cultures. Upon consulting several eminent bacteriologists on this subject, we found all of them favorably inclined toward conservative treatment of teeth, calling attention to the fact that organisms may just as frequently be cultured from glands and other tissues, not to speak of the almost positive liability of contamination in the procuring of specimens from the oral cavity.

### BONE REGENERATION

Bone, as we know, is a tissue which is changing as constantly as any other tissue of the body and under favorable conditions it is to be expected to grow and regenerate in order to supply the needs, deficiencies or repair indicated.

Claims have been made that this regenerated periapical bone is ''pathological bone''—or the result of a condensing osteitis, implying thereby that its quality is poor and that it is either already infected or is supersensitive to infection because of the fact that this bone is not properly nourished. These claims have been paraded under the name of osteo-sclerosis until the word has become a scarecrow to the profession. A very intimate study of this prenomenon has been brought out by Dr. Theo. H. Bast, Department of Anatomy, State University of Wisconsin, whose opinion is as follows:

BAST'S VIEWS ON BONE SCLEROSIS

"Under an excessive degree of irritation or stimulation, a great calcium deposit occurs in the periapical area more rapidly than the osteogenetic buds can arrange the final structural formation of the tissue by the establishment of Haversian systems in the compact bone, or by the proper excavation in the formation of true vascular cancellous bone."

Dr. Bast thus very clearly states that this substance which we have been warned against as being "sclerotic bone" is preosseous tissue which, when excavated by osteogenetic buds, becomes highly vital and perfectly normal vascular bone, and the sclerotic tissue, so-called, at the ends of the roots of teeth is only an intermediate step in bone development. (Fig. 7.)

In concluding we would like to emphasize the fact that inasmuch as teeth and their surrounding tissues are components of the human anatomy, it behooves us to make a careful examination of the general physical condition of each individual before deciding upon local treatment.

The principles involved in carefully selected root resections, include the first laws of septic surgery, viz., opening, removal of cause and débris, drainage and controlled healing. That this operation is a further aid to Nature has been proven time and again by the fact that there is complete regeneration of bone through the originally diseased area; that the function of the tooth is not hampered in any degree, and that radiographic and clinical evidences, both local and general, are negative.

We must consider these beautifully regenerated areas of bone as normal tissue until they are proven otherwise by a 100 per cent. effective technic without contamination and by further research. To date, in our opinion, this has not been done.—*Dental Items of Interest.*

